# Comparison of Fluoride Uptake Level by Enamel from Iranian School Brand and Standard NaF Mouthrinses

**DOI:** 10.5681/joddd.2009.002

**Published:** 2009-03-16

**Authors:** Gasem Ansari, Mojtaba Vahid Golpaygani, Ali Reza Sadr

**Affiliations:** ^1^Associate Professor, Department of Pedodontics, Faculty of Dentistry, Shahid Beheshti University of Medical Sciences, Tehran, Iran; ^2^Visiting staff, Faculty of Dentistry, Shahid Beheshti University of Medical Sciences, Tehran, Iran

**Keywords:** Enamel biopsy, fluoride, mouthrinse, NaF, uptake

## Abstract

**Background and aims:**

The use of fluoride mouthrinses has been proved to be one of the most effective ways to prevent tooth decay. A community based program using F+ rinse at school has also proved to be well-controlled and efficient. The aim of this investigation was to evaluate fluoride uptake level of a locally prepared NaF rinse used in Iran’s school program during 2005.

**Materials and methods:**

A total of 30 freshly extracted sound human premolars were collected and divided into two groups of 15. Each tooth then underwent two steps of sectioning; first the root was amputated from CEJ and then a longitudi-nal section was performed in bucco-lingual direction to provide two similar samples of the same tooth. A specific hemi-circular area on either of the experimental halves was treated by NaF rinse from USA or a locally prepared NaF used in school programs. Two subsequent biopsies were taken from each half using acid etch enamel biopsy technique. Fluoride and calcium content of the specimens were measured in order to evaluate fluoride uptake level and biopsy depth effect, respectively. Col-lected data were recorded in the forms provided and statistical analysis, mostly descriptive, was performed for comparison.

**Results:**

Based on the data collected, it appears that the use of F+ rinse would clearly improve enamel quality by a rise in fluoride concentration. Statistical analysis using a paired t-test and repeated measure method revealed that with 95% confi-dence fluoride concentration increases at both levels of biopsy with no statistically significant differences between the samples treated with two rinses.

**Conclusion:**

There seem to be reasonable potential for the clinical use of Iranian brand fluoride mouthrinse. There was no significant difference between the level of uptake from NaF from USA and the Iranian product in 2 layers of enamel biopsy.

## Introduction

It is widely believed that fluoride ion, in the form of mouthrinse, has a high potential for dental caries prevention when it is used on a daily or weekly basis in children. ^1,2^ Several studies have revealed the clinical efficacy of regular use of fluoride products, mouthwashes in particular, in reducing ever-increasing rate of tooth decay in almost every society.^3,4^ The frequency of fluoride mouthrinse application in schools has differences within communities with US schools having the weekly concentration once a week while European schools have it once every two weeks.^4^ Original concentration has been designed and packed in two different types of daily and weekly concentrations, with the latter having a higher concentration. However, it is believed that since caries is a multi-factorial entity and preventive measures are very much dependent on regular use for maximum efficacy, the concentration has no significant effect on the final result. Daily rinses have low potency and high frequency while weekly products are set with high potency and low frequency.^5,6,7^ Earlier reports have indicated that the concentration used in schools is mostly the weekly dose so as to ease the use at school with minimum educational interference.^5-7^ Kawasaki and Inaba ^8^ reported that a reduction of NaF concentration in mouthrinse products from 0.05% to 0.025% does not have a significant effect on the level of fluoride uptake by enamel. Generally, a superiority is considered for fluoride uptake in low-potency high-frequency method of use.^3^ To assess the efficacy of these techniques, several methods have been employed, including acid etch enamel biopsy, enamel micro-hardness, confocal laser scanning, quantitative transversal micro-radiography, and iodine permeability.^9,10^ The fluoride uptake level of any oral health product has been an obligatory requirement of FDA approval since 1995. Since then, several in vitro, in situ, and even in vivo trials have been conducted for different products including mouthrinses and their effect on fluoride uptake using Acid Etch Enamel Biopsy Technique.^11^ The depth of biopsy is calculated by the level of calcium in enamel in each specimen. This is followed by a potentiometery procedure using a fluoride-sensitive electrode to assess the level of fluoride ion.^12,13^ Kohli^14^ reported that enamel biopsy technique is a very sensitive technique for assessing the level of fluoride uptake by enamel. The aim of this in vitro investigation was to evaluate the fluoride uptake level of enamel from an Iranian fluoride mouthrinse used in school programs.

## Materials and Methods

A total of 30 sound freshly extracted human premolars were divided into two case and control groups in this in vitro study. Collected teeth were from patients aged 12-14 and extractions had been carried out for orthodontic reasons. Each sample tooth was assessed thoroughly both in a clinical setting and under a light microscope for their soundness. Teeth with any cracks, defects, white spots or restorations were excluded from the study. Each tooth was separately placed in a coded container of deionised water. Sample teeth were then subjected to two sectioning procedures, the first of which was horizontal to eliminate the root bulk from CEJ, while the second was longitudinal in bucco-lingual direction to provide two similar parts. One half was used as case and the other as control for a complete inter-group matching of the specimens. Two mouthrinses used were NaF rinse from USA (Stone Pharmaceuticals, Philadelphia, USA) and a locally prepared NaF (Shahrdaru, Tehran, Iran) used in school programs. The teeth were carefully covered with two layers of nail varnish leaving a window with a diameter of 6 mm on the enamel surface open for fluoride effect. A measure of 10 mL of each fluoride rinse was distributed among the empty containers of case or control groups. Samples were then placed into their corresponding coded containers for one hour in an attempt to ensure submergence of the samples in the fluoride rinse. A thorough rinse using distilled water was performed prior to enamel biopsy procedure. One mL of perchloric acid (0.5 mol) was used for enamel biopsy. New containers with this acid were prepared and the sample teeth were then placed into the containers. Two separate samples from each case were taken after every 30 seconds of exposure of the samples to acid. Samples taken were then immersed in 0.2 mol of KOH solution in order to reach an acid-base condition at 3 mL total. Containers with enamel biopsies were then subjected to a potentiometery procedure in which ion concentrations were measured. To achieve logical figures from the extracted data, a log was calculated using the following equation:

**Figure:**
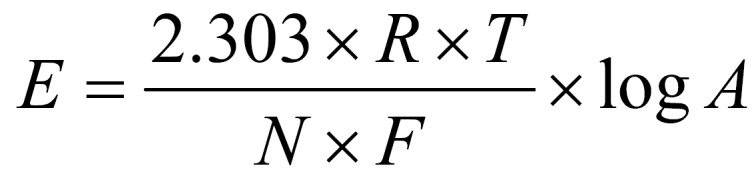


In the above-mentioned equation, E represents differences between A electrode (F^+^ specific) and B electrode (reference), R is gas fixed rate, F is fixed Farad, and A represents ion concentration. Calibration was made as standard with 0.01 ppm accuracy. A pH-meter was employed to assess the pH of sample liquids. Repeated measurements were made to reassure accuracy. Atomic absorption spectrophotometry was employed to assess the amount of Ca^++^and F^+^ ions in each sample container. Collected data was then transformed to ppm in order to facilitate interpretation of findings at enamel level.

## Results

The depth of the first and the second layer of enamel biopsy were 14.45 µm and 20.35 µm, respectively. This depth is advocated as being enough for measuring the F^+^ uptake by enamel.^15^ There were no statistically significant differences between the two groups of rinses using a paired t-test (P > 0.05). The mean F^+^ level at the first biopsy was measured at 1304.23 and 1898.78 ppm in the case and control groups of Iranian fluoride rinse, respectively (Table 1). The mean F^+^ level at first biopsy was measured at 1293.56 and 2074.97 ppm in the case and control groups of USA F^+^ rinse, respectively.

**Table 1:** Mean (SD) level of fluoride ion measurements at the first layer biopsy of the two brands evaluated

Mouthrinse	F^+^ level in the control group (ppm)	F^+^ level in the experimental group (ppm)
Iranian Brand (A)	1304.23 (357.29)	1898.78 (437.21)
USA Brand (B)	1293.56 (342.78)	2074.97 (593.38)

Statistical analysis of data revealed significant differences between the control and experimental groups of each mouthrinse (P < 0.05) using paired t and Wilkoxson tests. Repeated measure test revealed no significant differences between the groups of the two mouthrinses (P > 0.05).

**Figure 1:**
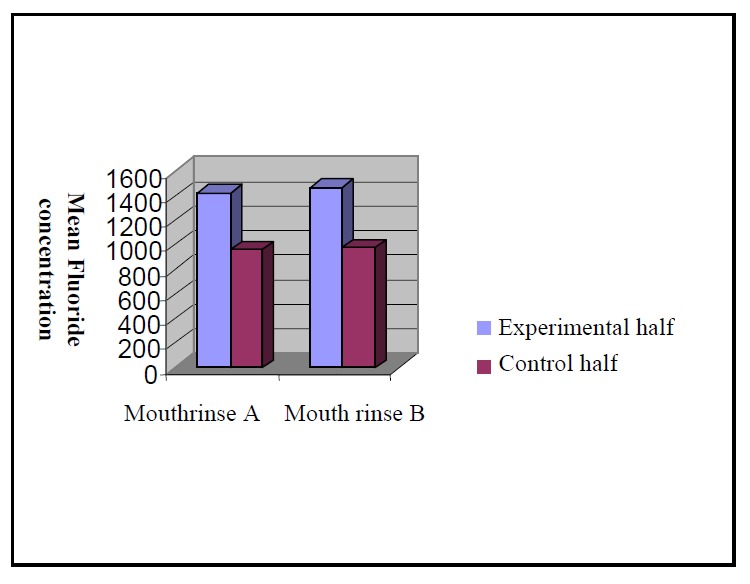
Mean fluoride level measurements in first layer of biopsy.

Mean F^+^ measured in the first layer of biopsy were 964.57 and 1427.90 for control and experimental groups of Iranian rinse, respectively (Figure1). These figures were 984.70 and 1473.43 ppm for F^+^ level of the second biopsy in USA rinse, respectively.

Statistical analysis using a paired t-test revealed a significant difference between the level of F^+^ measurements between the control and experimental halves of the teeth in both rinse groups. However, the differences between the two rinse groups were not significant.

**Figure 2:**
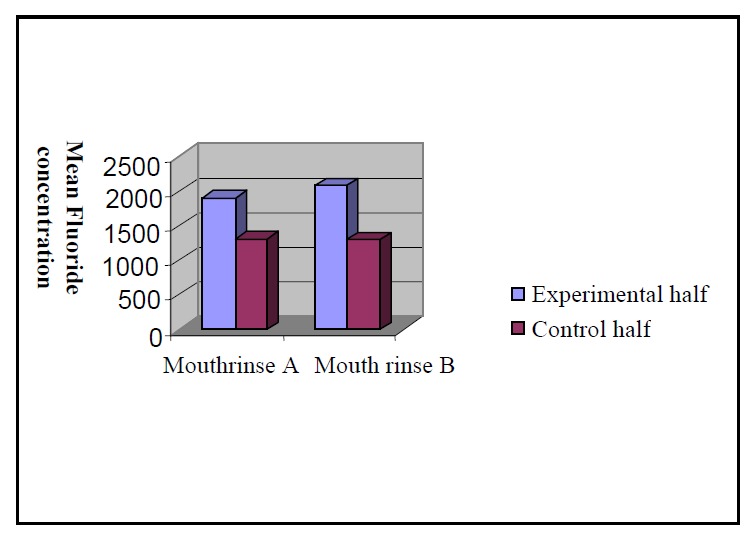
Mean fluoride level measurements in second layer of biopsy.

A further comparison between the two layers using the same test results revealed no significant differences of uptake in the deeper layer (Table 2,Figure 2).

**Table 2:** Mean (SD) level of fluoride ion measurements at the second layer biopsy of the two brands evaluated

Mouthrinse	F^+^ level in the control group (ppm)	F^+^ level in the Experimental group (ppm)
Iranian Brand (A)	946.57 (339.45)	1427.90 (327.45)
USA Brand (B)	984.70 (323.55)	1473.43 (399.38)

## Discussion

WHO recommendations suggest that the use of fluoride mouthrinses is an efficient preventive tool to maintain good oral health in young communities of different societies.^11,16^ Iran’s National Plan for fluoride mouthrinse at schools was first introduced in 2001 by the Ministry of Health and Medical Education in collaboration with WHO regional branch (EMRO). A new approach, however, requires to be tested for its effectiveness: in this case its effect on reducing caries risk by an increase in F^+^ level of treated enamel. In order to carry out such measurements, an evaluation path was adopted using Acid Etch Enamel Biopsy Technique based on similar studies in the past.^14,17,18^ It is believed that enamel biopsy provides a direct measurement as one of the most reliable techniques for measuring F^+^ level of enamel.^19,20^ Root and Schreiber^20^ reported that long-term use of fluoride rinse has a greater chance of F^+^ uptake by enamel than short periods, indicating a more reliable preventive role when fluoride rinse is used regularly. It is worth pointing out that the use of enamel biopsy, which was first introduced by McCann,^12^ has frequently been reported to be used by many researchers who have employed 0.5 mol perchloric acid for 30 seconds. This provides 10 biopsies from surface enamel sufficient for further measurements.^12^ In the meantime, several studies have employed the technique for two consecutive times in order to achieve a deeper sample for a more accurate and precise reading with no significantly different results. A lower concentration was reported as expected.^13,14,21^ It is also suggested that the vibration of specimens within the acid liquid will prevent the return of released F^+^ into enamel bulk.^15^ The mean F^+^ uptake from the control and experimental groups of both mouthrinses were found to be at 594.5 ppm and 781.4 ppm, respectively. These figures were close to the findings of Kohli et al^14^ with 578 ppm and Mellberg et al^19^ with 563 ppm in the same depth of biopsy.

## Conclusion

Based on the statistical analysis performed, these data did not represent any significant differences between the Iranian and US brands of NaF; however, a significant difference was found between fluoridated and non-fluoridated samples as further confirmation of earlier reports.
